# Changes in cerebral oxygen saturation and cerebral blood flow velocity under mild +Gz hypergravity

**DOI:** 10.1152/japplphysiol.00119.2019

**Published:** 2019-06-06

**Authors:** Toru Konishi, Takuya Kurazumi, Tomokazu Kato, Chiharu Takko, Yojiro Ogawa, Ken-ichi Iwasaki

**Affiliations:** ^1^Department of Social Medicine, Division of Hygiene, Nihon University School of Medicine, Tokyo, Japan; ^2^Aeromedical Laboratory, Japan Air Self-Defense Force, Ministry of Defense, Saitama, Japan

**Keywords:** artificial hypergravity, human centrifuge, near-infrared spectroscopy, transcranial Doppler ultrasonography

## Abstract

We previously reported that cerebral blood flow (CBF) was reduced by even mild +Gz hypergravity. Regional cerebral oxygen saturation as measured by near-infrared spectroscopy (C-rSO_2_) has been widely used to detect cerebral ischemia in clinical practice. For example, decreases in C-rSO_2_ reflect reduced CBF or arterial oxygen saturation. Thus it was hypothesized that C-rSO_2_ would decrease in association with reduced CBF during mild hypergravity. To test this hypothesis, we measured CBF velocity by transcranial Doppler ultrasonography and C-rSO_2_ during mild +Gz hypergravity while participants were in a sitting position. Among 17 male participants, 15 completed 21 min of exposure to +1.5 Gz generated by short-arm centrifuge. C-rSO_2_ and mean CBF velocity in the middle cerebral artery (MCBFV_MCA_) during centrifugation were averaged every 5 min and compared with pre-hypergravity (+1.0 Gz). C-rSO_2_ did not change significantly throughout centrifugation, although MCBFV_MCA_ gradually decreased from the beginning (−1.2% at 0–5 min), and significantly decreased at 5–10 min (−4.8%), 10–15 min (−6.7%), and 15–20 min (−7.4%). Contrary to our hypothesis, decreases in C-rSO_2_ were not detected, despite reductions in CBF velocity during hypergravity. Since some assumptions, such as unaltered arteriovenous volume ratio, hemoglobin concentration, extracranial blood flow, and brain activity, need to be satisfied to monitor cerebral ischemia by C-rSO_2_, the present results suggest that these necessary assumptions for near-infrared spectroscopy are not always applicable, and that cerebral oxygenation may not precisely reflect decreases in CBF under mild +Gz hypergravity.

**NEW & NOTEWORTHY** To our knowledge, this is the first study to evaluate simultaneously cerebral oxygenation monitored by near-infrared spectroscopy and cerebral blood flow (CBF) monitored by transcranial Doppler under +1.5 Gz hypergravity. Contrary to our hypothesis, there was no significant correlation between CBF velocity and regional cerebral oxygen saturation (C-rSO_2_). However, an incomplete case nearly involving syncope suggests the possibility that C-rSO_2_ can detect a remarkable decrease in CBF with development of presyncope during +Gz hypergravity.

## INTRODUCTION

Humans are exposed to hypergravity in various situations, such as during launchings/landings of spacecraft and while performing aerial maneuvers in high-performance aircraft, riding roller coasters, and participating in motorsports. The physiological impacts of hypergravity depend on various characteristics, such as direction, magnitude, onset rate, and/or sustained time (33). On the other hand, it has been proposed that intermittent exposure to mild +Gz (head-to-foot) hypergravity in a human centrifuge could help prevent or mitigate spaceflight-induced physiological deconditioning (7, 10). Understanding the physiological changes experienced by humans during exposure to mild +Gz hypergravity is therefore important for understanding various situations, as well as for the future implementation of human centrifuges (10). We previously reported that cerebral blood flow (CBF) as measured by transcranial Doppler ultrasonography (TCD) was significantly reduced under even mild +Gz hypergravity, and did not lead to any changes in arterial blood pressure (ABP) at heart level (15, 20, 24).

Cerebral oxygenation as measured by near-infrared spectroscopy (NIRS) has been clinically used to monitor cerebral ischemia (3, 14, 19). For example, decreases in cerebral oxygenation indexes are thought to reflect reduced CBF, hemoglobin concentration, or arterial oxygen saturation (SaO_2_). Thus it was hypothesized that cerebral oxygenation would decrease in association with reduced CBF under mild +Gz hypergravity. However, to our knowledge, no study has been conducted to evaluate simultaneously changes in CBF and cerebral oxygenation under +Gz hypergravity. Therefore, to test this hypothesis, we evaluated changes in regional cerebral oxygen saturation as measured by NIRS (C-rSO_2_) during 21 min of +1.5 Gz centrifugation and compared the results with those in CBF velocity as measured by TCD.

## METHODS

### 

#### Participants.

The entire study protocol was approved by the Institutional Review Board of Nihon University School of Medicine (No. 29–2-0; 4 July 2017) and registered in the University Hospital Medical Information Network (UMIN) clinical trial registry (ID: UMIN000028466). The study procedures adhered to the tenets of the Declaration of Helsinki. In total, 17 healthy male volunteers who had no prior experience with a human centrifuge participated in the study (age 24 ± 1 yr; height 172.6 ± 6.6 cm; weight 69.2 ± 7.7 kg; mean ± SD). All participants provided written informed consent and were screened based on their medical history and a physical examination, including an electrocardiogram and ABP measurement. In addition, in all participants it was confirmed that CBF velocity signals in the middle cerebral artery (MCA) could be obtained by TCD. All participants fasted for ≥2 h before the experiments, and refrained from engaging in heavy exercise or consuming caffeinated or alcoholic beverages for at least 12 h before the experiments.

#### Equipment.

The short-arm human centrifuge (Daiichi Medical, Tokyo) at Nihon University was used in the present study. The experimental room where the centrifuge is located was environmentally controlled at an ambient temperature of 23~25°C. A gimbaled cabin was attached to the end of a rotating arm with a radius of 1.7 m. The participants were seated facing outside the cabin and instructed to minimize their head movement. The top of the cabin reclined toward the center during centrifugation. The resultant force, which was a combination of the Earth’s gravitational force and the centrifugal force, was directed along the participant’s longitudinal (head-to-foot) axis. To limit the visual stimuli and prevent nausea during centrifugation, the cabin door was closed so that the participant could not see outside.

A three-lead electrocardiogram and SaO_2_ by pulse oximetry (SpO_2_) were monitored (Life Scope PT, BSM-1763; Nihon Kohden, Tokyo). Regional oxygen saturation (rSO_2_) was measured using two probes on an NIRS module (INVOS SPS; Covidien, Mansfield, MA), and the data were sent to a Life Scope TR monitor (BSM-6301, Nihon Kohden). One probe was placed on the right side of the forehead to measure C-rSO_2_ and the other was placed on the left upper arm at heart level to measure peripheral rSO_2_ (P-rSO_2_). Partial pressure of expiratory carbon dioxide (CO_2_) was monitored by an infrared CO_2_ sensor (OLG-2800; Nihon Kohden). Continuous ABP in the left middle finger was measured, and brachial ABP at heart level was obtained by subtracting the hydrostatic pressure between the finger and the heart using a height sensor (Finometer MIDI; Finapres Medical Systems, Amsterdam, The Netherlands). Continuous CBF velocity in the MCA was measured by TCD with a 2-MHz probe placed over the right temporal window (EZ-Dop; Compumedics Germany, Singen, Germany). The probe was fixed at a constant angle with a probe holder that was individually customized to fit the facial bones and ear structures of each participant just before data measurement.

Commercial software (Notocord-hem 4.3.0.74; Notocord, Paris, France) was used to record waveforms of the electrocardiogram, ABP, CBF velocity, and expiratory CO_2_ with a 1-kHz sampling rate. The SpO_2_, C-rSO_2_, and P-rSO_2_ data were recorded using the Life Scope BSM-1763 with a 0.3-Hz sampling rate. Dedicated software (BSM PC-Viewer; Nihon Kohden) was used to extract these data.

#### Protocol.

Pre-hypergravity (+1.0 Gz) data were collected from the participants before centrifugation for 6 min after ≥15 min of quiet rest in an upright sitting position in the cabin of centrifuge. The participants were then exposed to mild hypergravity (+1.5 Gz) generated by the centrifuge. The centrifugation was kept at 24.3 rpm for 21 min to generate +1.5 Gz at heart level. The onset and offset rates were +0.5 G/min and –0.1 G/min, respectively. Mild hypergravity (+1.5 Gz) data were collected for 21 min of +1.5 Gz centrifugation. Waveforms of the electrocardiogram, ABP, CBF velocity, and expiratory CO_2_ were continuously monitored by the doctors during centrifugation. In addition, a charge-coupled device camera was installed in the cabin to monitor the conditions of both the on-board participant and the inside of the cabin. Moreover, the on-board participant and the doctor who was in charge of operating the centrifuge could have a conversation via an intercom. However, participants who completed the scheduled protocol did not have conversations during centrifugation. If any signs and/or symptoms of suspected presyncope, such as nausea, sweating, gray-out, bradycardia, or hypotension, were observed, the centrifugation was terminated. In the present study, 15 participants completed the scheduled centrifuge protocol, but 2 could not because of the development of strong nausea accompanied by abnormal vital signs.

#### Data analysis.

Mean CBF velocity in the MCA (MCBFV_MCA_) and mean ABP at heart level (MAP_heart_) were obtained from each continuous waveform of CBF velocity and ABP on a beat-by-beat basis. The distance between the heart and the position where the TCD probe was placed was measured to calculate the hydrostatic pressure between heart and MCA level. Hydrostatic pressure was estimated as the measured distance (in cm) multiplied by 0.78 mmHg at +1.0 Gz or 1.17 mmHg at +1.5 Gz, assuming that the specific gravity of mercury at 37°C (density 13,500 kg/m^3^) referenced to 37°C water (density 993 kg/m^3^) is 13.6, and the specific gravity of whole blood at 37°C referenced to 37°C water is 1.06 (35). Mean ABP at the MCA level (MAP_MCA_) was then estimated by subtracting hydrostatic pressure from MAP_heart_. Heart rate (HR) was calculated on a beat-by-beat basis from the R-R interval obtained from the electrocardiogram continuous waveform. End-tidal CO_2_ (ET_CO2_) was obtained from the expiratory CO_2_ continuous waveform. The SpO_2_, C-rSO_2_, and P-rSO_2_ data were extracted with a 1-Hz sampling rate.

To evaluate the time course of changes in the measured variables, the initial 5 min of 6-min pre-hypergravity (+1.0 Gz) data were used as a pre-hypergravity data segment. Mild hypergravity (+1.5 Gz) data during the 21-min centrifugation period were divided into the following four data segments by 5-min intervals from the point at which centrifugation reached 24.3 rpm (+1.5 Gz at heart level): 0–5 min, 5–10 min, 10–15 min, and 15–20 min. A total of five data segments (a pre-hypergravity data segment and 4 hypergravity data segments) were used for the analysis. Five-minute averages for MCBFV_MCA_, C-rSO_2_, P-rSO_2_, MAP_heart_, MAP_MCA_, HR, SpO_2_, and ET_CO2_ were obtained by averaging data during each 5-min data segment.

#### Statistical analysis.

All statistical analyses were performed using R (The R Foundation for Statistical Computing, Vienna, Austria). Data are shown as means ± SD. Values of *P* < 0.05 were considered statistically significant. Normality was evaluated by the Kolmogorov-Smirnov test. For the variables with a normal distribution, one-way repeated-measures analysis of variance (ANOVA) was performed with data segment (pre-hypergravity, 0–5 min, 5–10 min, 10–15 min, and 15–20 min) as a factor, followed by Holm’s post hoc test (paired *t*-test with the *P* value adjusted by Holm’s method) for multiple comparisons. If the sphericity assumption was violated by Mauchly’s test in the ANOVA, the Greenhouse-Geisser correction was used to adjust the degrees of freedom. Therefore, for the variables for which sphericity were violated, the degrees of freedom (df) was not an integral number. For the variables that were not normally distributed, Friedman tests were performed with data segment as a factor, followed by Holm’s post hoc test (Wilcoxon signed-rank test with the *P* value adjusted by Holm’s method) for multiple comparisons. These statistical analyses were performed using EZR (Saitama Medical Center, Jichi Medical University, Saitama, Japan; https://cran.r-project.org/web/packages/RcmdrPlugin.EZR/), which is a graphical user interface for R (16). To evaluate the relationship between C-rSO_2_ and MCBFV_MCA_, the repeated-measures correlation analysis first introduced by Bland and Altman (5) was performed using the rmcorr R package developed by Bakdash and Marusich (https://cran.r-project.org/web/packages/rmcorr/) (4).

## RESULTS

**Fig. 1.**
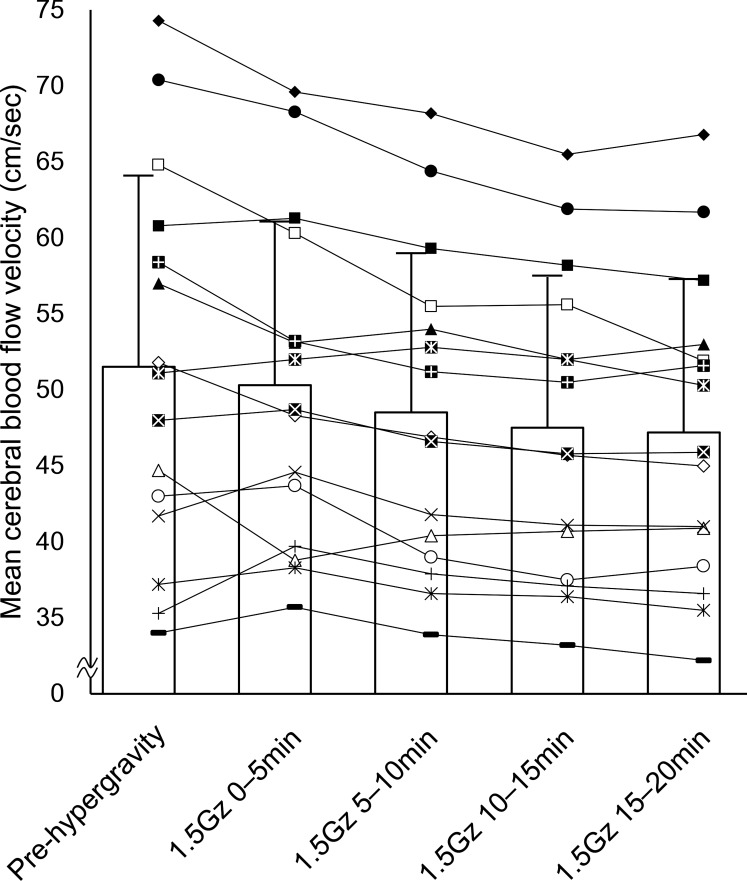
Time course of changes in mean cerebral blood flow velocity in the middle cerebral artery before and during +1.5 Gz centrifugation for all participants who completed the study protocol. Pre-hypergravity: average of pre-hypergravity 5-min sections (+1.0 Gz); 0–5 min, 5–10 min, 10–15 min, and 15–20 min: 5-min averages of the 0–5-min, 5–10-min, 10–15-min, and 15–20-min data segments during +1.5 Gz centrifugation. Lines with markers represent the changes in mean cerebral blood flow velocity in the middle cerebral artery during each data segment for each participant (*n* = 15). White bars with error bars represent the average values and SDs of the 15 participants.

**Fig. 2.**
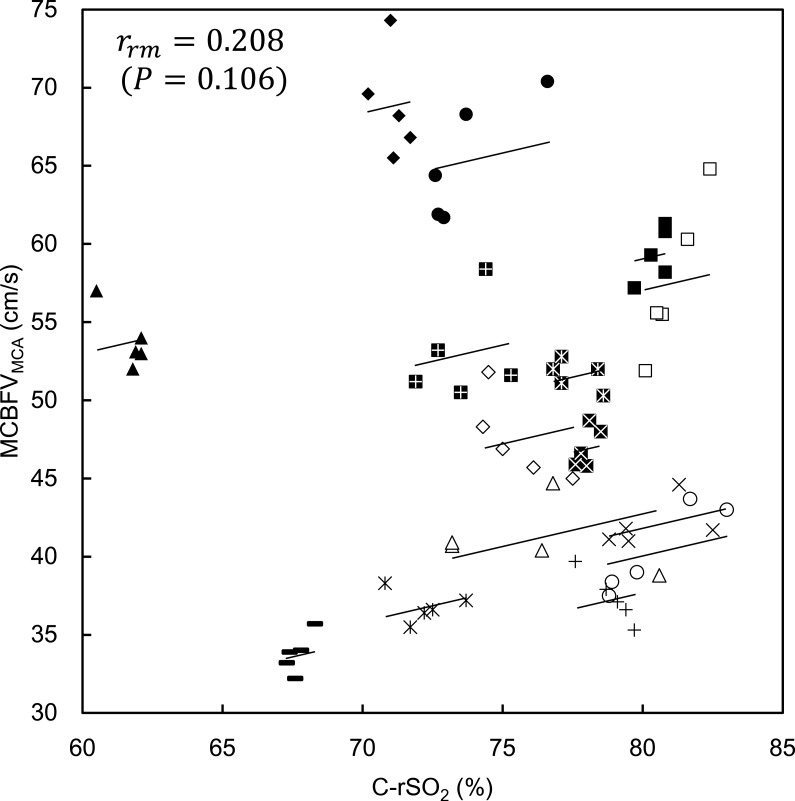
Repeated-measures correlation between regional cerebral oxygen saturation (C-rSO_2_) and mean cerebral blood flow velocity in the middle cerebral artery (MCBFV_MCA_). Each marker represents the measured values of each participant, and the total of 75 data points (5 data segments × 15 participants who completed the task) are shown. Solid lines represent the regression line of each participant. The value of the repeated measures correlation coefficient (*r*_rm_) is shown with the *P* value.

For the group averages, data from 15 participants who completed 21 min of exposure (age 24 ± 1 yr; height 172.5 ± 6.8 cm; weight 69.7 ± 7.7 kg; mean ± SD) were used. Table 1 shows the 5-min averages of measured variables in each of the five data segments: one pre-hypergravity and four data segments during +1.5 Gz centrifugation (0–5 min, 5–10 min, 10–15 min, and 15–20 min). A significant main effect of data segment was found in MCBFV_MCA_ [*F*(1.83,25.73) = 15.18, *P* < 0.001 (ANOVA)]. MCBFV_MCA_ tended to decrease from the beginning of centrifugation, but MCBFV_MCA_ at 0–5 min did not reach statistical significance compared with pre-hypergravity (−1.2%). Then, MCBFV_MCA_ significantly decreased at 5–10 min (–4.8%), 10–15 min (−6.7%), and 15–20 min (−7.4%). However, no significant difference was found between 10–15 min and 15–20 min. Figure 1 shows the changes in MCBFV_MCA_ for all individual participants who completed the scheduled centrifugation. C-rSO_2_ [*F*(1.82,25.49) = 1.98, *P* = 0.160 (ANOVA)] showed almost no change (−1.0% at 15–20 min). P-rSO_2_ [*F*(2.27,31.89) = 0.50, *P* = 0.632 (ANOVA)] did not change significantly throughout centrifugation. Figure 2 shows the repeated-measures correlation between C-rSO_2_ and MCBFV_MCA_. The correlation coefficient (*r*_rm_) value was 0.208 (df = 59, 95% confidence interval [–0.05,0.44], *P* = 0.106).

**Table 1.** Five-minute averages of measured variables before and during +1.5 Gz centrifugation

		+1.5 Gz				
	+1.0 Gz Pre-hypergravity	0–5 min	5–10 min	10–15 min	15–20 min	*P* value
MCBFV_MCA_, cm/s	51.5 ± 12.5	50.3 ± 10.7	48.5 ± 10.4*††	47.5 ± 9.9**††‡‡	47.2 ± 10.0**††‡‡	<0.001 (A)
C-rSO_2_, %	75.9 ± 6.0	75.3 ± 5.8	74.8 ± 5.2	74.8 ± 5.3	75.0 ± 5.2	0.160 (A)
P-rSO_2_, %	79.5 ± 4.6	79.6 ± 4.8	79.0 ± 4.9	79.6 ± 5.2	79.6 ± 5.3	0.632 (A)
MAP_heart_, mmHg	79.9 ± 13.9	86.3 ± 11.4**	86.4 ± 10.8*	86.8 ± 10.4**	88.0 ± 10.8***	<0.001 (F)
MAP_MCA_, mmHg	56.1 ± 14.3	50.6 ± 12.1**	50.8 ± 11.7*	51.1 ± 11.3	52.3 ± 11.6	0.001 (F)
HR, beats/min	65.0 ± 9.0	71.8 ± 8.6***	72.1 ± 9.6***	72.6 ± 9.4***	73.4 ± 10.0***	<0.001 (A)
SpO_2_, %	98.0 ± 1.0	98.7 ± 0.8*	98.1 ± 0.9†††	98.0 ± 0.9††	98.0 ± 0.9††	<0.001 (A)
ET_CO2_, Torr	39.0 ± 2.5	35.4 ± 2.9***	34.8 ± 2.8***	34.8 ± 2.9***	34.4 ± 2.7***†	<0.001 (A)

Values are means ± SD. Pre-hypergravity: average of pre-hypergravity 5-min sections (+1.0 Gz); 0–5 min, 5–10 min, 10–15 min, and 15–20 min: 5-min averages of the 0–5-min, 5–10-min, 10–15-min, and 15–20-min data segments during +1.5 Gz centrifugation. MCBFV_MCA_: mean cerebral blood flow velocity in the middle cerebral artery; C-rSO_2_: regional cerebral oxygen saturation; P-rSO_2_: regional oxygen saturation at heart level (upper arm); MAP_heart_: mean arterial pressure at heart level; MAP_MCA_: mean arterial pressure at the middle cerebral artery level; HR: heart rate; SpO_2_: peripheral arterial oxygen saturation; ET_CO2_: partial pressure of end-tidal carbon dioxide. *P* values are expressed as one-way repeated-measures analysis of variance with data segment as a factor (A), or Friedman tests with data segment as a factor (F).

* *P* < 0.05,

** *P* < 0.01,

*** *P* < 0.001 (*P* value of Holm’s post hoc test compared with the pre-hypergravity data segment);

† *P* < 0.05,

†† *P* < 0.01,

††† *P* < 0.001 (*P* value of Holm’s post hoc test compared with the 0–5-min data segment);

‡‡ *P* < 0.01 (*P* value of Holm’s post hoc test compared with the 5–10-min data segment).

MAP_heart_ significantly increased throughout centrifugation compared with pre-hypergravity [χ^2^ = 24.765, df = 4, *P* < 0.001 (Friedman)]. On the other hand, MAP_MCA_ during centrifugation was lower than that during pre-hypergravity (–9.2% at 0–5 min, –8.6% at 5–10 min, –7.8% at 10–15 min, and –5.7% at 15–20 min), and statistical significance was found at 0–5 min and 5–10 min [χ^2^ = 18.268, df = 4, *P* = 0.001 (Friedman)]. HR significantly increased throughout centrifugation compared with pre-hypergravity [*F*(2.20,30.87) = 26.777, *P* < 0.001 (ANOVA)]. SpO_2_ slightly but significantly increased compared with pre-hypergravity at 0–5 min, and returned to pre-hypergravity levels after 5–10 min [*F*(2.58,36.25) = 10.04, *P* < 0.001 (ANOVA)]. ET_CO2_ significantly decreased compared with pre-hypergravity throughout centrifugation [*F*(4,56) = 77.48, *P* < 0.001 (ANOVA)].

**Fig. 3.**
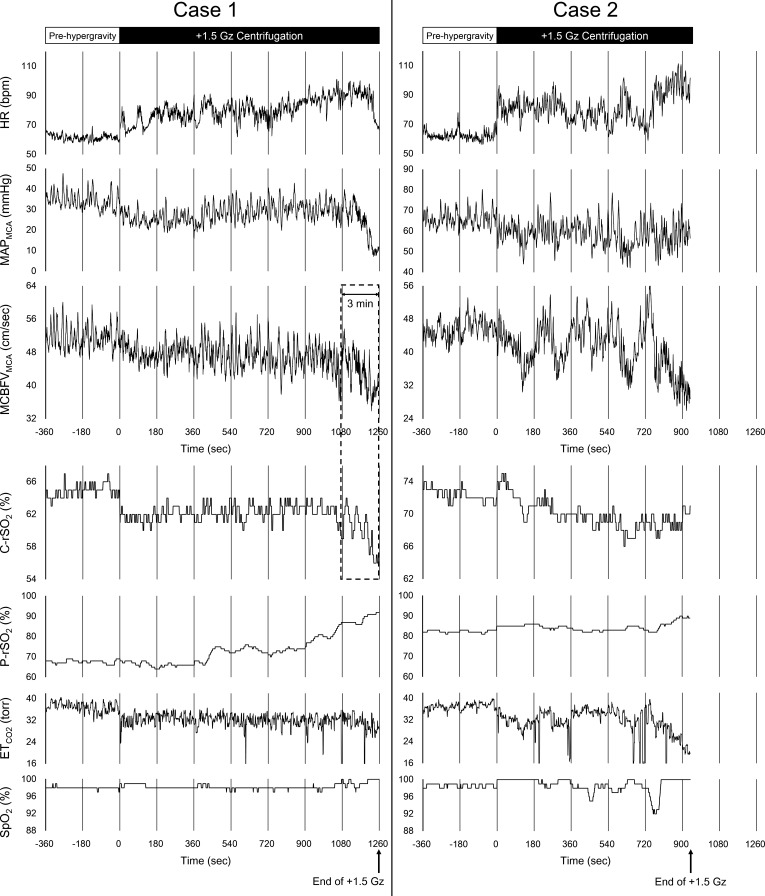
Time courses of changes in measured variables for 2 participants whose centrifugation was terminated. Left-side charts show time courses of changes in measured variables for *case 1*, and right-side charts show those for *case 2*. From the top, each column shows the time courses of changes in heart rate (HR), mean arterial pressure at the middle cerebral artery level (MAP_MCA_), mean cerebral blood flow in the middle cerebral artery (MCBFV_MCA_), regional cerebral oxygen saturation (C-rSO_2_), regional peripheral (upper arm at heart level) oxygen saturation (P-rSO_2_), partial pressure of end-tidal carbon dioxide (ET_CO2_), and peripheral arterial oxygen saturation (SpO_2_). Values for HR, MAP_MCA_, and MCBFV_MCA_ were plotted on a beat-by-beat basis. Values for C-rSO_2_, ETCO_2_, P-rSO_2_, and SpO_2_ were plotted with a 1-Hz sampling rate. The dotted box shows the last 3 min of +1.5 Gz hypergravity for *case 1*. In *case 1*, the elapsed time of +1.5 Gz centrifugation reached nearly 21 min, but was not fully completed.

Two participants could not complete 21 min of exposure because of strong nausea accompanied by abnormal vital signs. In one case, MAP_MCA_ rapidly decreased without decreases in HR (Fig. 3, *case 1*). Both MCBFV_MCA_ and C-rSO_2_ decreased simultaneously after ~18 min of exposure to +1.5 Gz hypergravity. These decreases (percent change from pre-hypergravity data) during the last 3 min of +1.5 Gz hypergravity were –16.2% and –7.5%, respectively (dotted box in Fig. 3), while those during the last 1 min were –24.3% and –11.2%, respectively. HR remained increased, but showed a sudden drop just before the deceleration (termination of +1.5 Gz exposure). In the other case, although both HR and MAP_MCA_ tended to increase, both MCBFV_MCA_ and ET_CO2_ rapidly decreased after ~12 min of exposure to +1.5 Gz hypergravity (Fig. 3, *case 2*). No obvious change in C-rSO_2_ was observed during this period. These cases were carefully monitored, and both recovered a short time after stopping the centrifuge. Neither of the participants had any past medical history or any significantly different background characteristics compared with the other participants.

## DISCUSSION

The aim of the present study was to test our hypothesis that C-rSO_2_ would decrease in association with a reduction in CBF during mild +Gz centrifugation by evaluating simultaneously changes in CBF and cerebral oxygenation. The results showed that MCBFV_MCA_ gradually decreased from the beginning of the +1.5 Gz centrifugation and reached statistical significance after a 5–10 min data segment. On the other hand, no significant change in C-rSO_2_ was detected throughout centrifugation. No significant correlation was found between MCBFV_MCA_ and C-rSO_2_. Contrary to our hypothesis, the results of the present study suggest that the changes in C-rSO_2_ did not precisely reflect the reduction in CBF under mild +Gz hypergravity in the participants who completed the exposure without any symptoms.

Monitoring of cerebral oxygenation as measured by NIRS has been widely used to detect cerebral ischemia, especially during cardiovascular surgery (3, 14, 19). Several assumptions, such as unaltered arteriovenous volume ratio, hemoglobin concentration, extracranial blood flow, and brain activity (6, 19, 28, 34, 38), need to be satisfied to monitor cerebral ischemia by cerebral oxygenation. Prior to the present experiment, we had assumed that the arteriovenous volume ratio at brain level would not change considerably during centrifugation. The internal jugular vein, as the main venous outflow from the brain, might collapse in the upright sitting position in most study participants (13); therefore, an increase in the gravitational force by +1.5 Gz hypergravity might change venous blood volume minimally in the brain. Also, we had assumed that hemoglobin concentration would not change during centrifugation. We had assumed that the impact of extracranial blood flow would be negligible because the NIRS-based oximetry used in the present study has multidistance light detectors and can subtract light absorption in superficial tissue from that in deeper tissue (32). Furthermore, it had been considered that brain activity would not change markedly in participants without presyncope under mild +Gz hypergravity. Thus we had believed that the necessary assumptions to monitor cerebral ischemia by cerebral oxygenation would be satisfied under mild +Gz hypergravity, and thus had hypothesized that C-rSO_2_ would decrease mainly in association with a reduction of CBF under mild +Gz hypergravity.

However, contrary to our hypothesis, no significant changes in C-rSO_2_ were detected throughout centrifugation, although MCBFV_MCA_ decreased significantly. There are several possible mechanisms for explaining the present results of unchanged C-rSO_2_ despite the reduced CBF. First, the arteriovenous volume ratio at brain level might change during centrifugation; this may be induced by decreases in cerebral venous blood volume due to the gravitational force during centrifugation. In some participants, an additional 0.5 gravitational force might drain venous blood through the noncollapsed internal jugular vein or the secondary veins of the vertebral, epidural, and deep cervical veins in the sitting position (2, 9) during hypergravity. In addition, the cerebral autoregulation system should dilate arterioles as resistance vessels to maintain CBF, mitigating the reduced arterial pressure in the MCA during some periods of hypergravity, as will be mentioned later. It is possible that the dilation of arterioles leads to a relative increase in the percentage of arterial blood volume despite decreases in CBF. The changes in the arteriovenous ratio should reduce the accuracy of C-rSO_2_ monitoring because the NIRS-based oximetry used in the present study measures oxygen saturation using a fixed arteriovenous volume ratio of 75% venous and 25% arterial (34). Second, hemoglobin concentration might increase because of plasma volume extravasation by the gravitational force (22) under hypergravity. Third, the NIRS-based oximetry used in the present study has been reported to be affected by extracranial contamination (17), so extracranial blood flow might change and affect the C-rSO_2_ value during centrifugation. Finally, brain activity might increase under even mild +Gz hypergravity, inducing increases in regional blood flow by vascular dilation to compensate for the increased oxygen demand (37). In fact, Smith et al. (31) reported that in participants who showed symptoms of presyncope, prefrontal cortex activity was increased by psychological stress during centrifugation (maximum of +1.4 Gz). Thus the assumptions needed to use cerebral oxygenation as measured by NIRS for detecting cerebral ischemia cannot be always satisfied during mild +Gz hypergravity. In fact, some studies have evaluated the changes in CBF by TCD and NIRS simultaneously. For example, good correlations between CBF velocity by TCD and NIRS-derived variables during cardiovascular surgery have been reported (14, 19); however, the response to head-up tilt has been controversial. Krakow et al. (21) reported the good followability of oxyhemoglobin and C-rSO_2_ to MCBFV_MCA_, but Canova et al. (8) reported finding no correlation between tissue hemoglobin index and MCBFV_MCA_. Thus the relationship between TCD and NIRS has not always been constant in head-up tilt studies (8, 21), which have used different durations, head-up tilt angles, and participant populations. It is therefore important to consider the details of the study protocol and conditions that minimize the violation of necessary assumptions of NIRS.

Although intermittent and repeated exposure to artificial hypergravity via a human centrifuge has been proposed as a countermeasure against spaceflight-induced physiological deconditioning (7, 10), in the present study, MCBFV_MCA_ was significantly decreased under even mild +Gz hypergravity, which was consistent with our previous reports (15, 20, 24). Hence, the decreases in CBF were thought to be one of the adverse effects during even mild centrifugation, suggesting that careful monitoring of CBF is needed during the exposure. However, several challenges remain for using TCD during centrifugation in a practical rather than an experimental setting, such as the difficulty in the fixation of the TCD probe at a constant angle without some specific methods, or the possibility of an inadequate temporal acoustic window in some individuals (23, 25). We had believed that alternative means would be needed to monitor CBF during centrifugation, and we had expected that the monitoring of cerebral oxygenation as measured by NIRS would be easily utilizable and applicable to everyone. To our knowledge, there have been no reports evaluating the changes in CBF under +Gz hypergravity by both NIRS and TCD simultaneously, but several studies have detected a reduction in CBF by NIRS under a “high” +Gz hypergravity environment in participants who showed symptoms of almost loss of consciousness (A-LOC) (27, 30). Ryoo et al. (27) reported an average −5.3% decrease in C-rSO_2_ by NIRS during A-LOC. In general, decreases in MCBFV_MCA_ are much larger during A-LOC. For example, Kawai et al. (18) reported an average −48% decrease in MCBFV_MCA_ by TCD during A-LOC. In the present study, one of the two incomplete cases was considered likely to be presyncope (Fig. 3, *case 1*). This case showed rapid and large decreases in both MCBFV_MCA_ (−16.2%) and C-rSO_2_ (−7.5%) during the last 3 min of +1.5 Gz hypergravity. Especially, the decreases in MCBFV_MCA_ and C-rSO_2_ reached to −24.3% and −11.2% in the last 1 min. The followability of C-rSO_2_ to the decreases in MCBFV_MCA_ was good during this period. Therefore, the present and previous results together suggest that reduced CBF during centrifugation can be detected by cerebral oxygenation only if the extent of decreases in CBF is large enough to develop presyncope and preponderate the impact of violating the necessary assumptions for NIRS.

In addition, remarkable increases in P-rSO_2_, for which the probe was placed on the left upper arm at heart level, were observed in the two incomplete cases (Fig. 3). It was presumed that these increases in P-rSO_2_ were due to sympathetic hyperactivity, which led to the dilation of cutaneous vessels induced by nitric oxide (NO) accompanying the release of acetylcholine in the sweat glands (39). On the other hand, no significant changes in P-rSO_2_ or C-rSO_2_ were detected in the complete cases. Therefore, P-rSO_2_ can be used as the index when paying attention to the development of abnormal symptoms during mild +Gz centrifugation.

Although both of the incomplete cases showed rapid decreases in MCBFV_MCA_ during the last few minutes before the termination of the study protocol, the mechanisms inducing the decreases in CBF seemed to differ. In one case (Fig. 3, *case 1*), which was similar to an orthostatic hypotension patient, MAP_MCA_ remarkably decreased during the last few minutes of centrifugation; in association, MCBFV_MCA_ also decreased. Furthermore, HR suddenly dropped just before the termination of exposure to +1.5 Gz, suggesting that the vasovagal reflex was induced. It was thought that this participant almost fell into syncope and the decreases in MCBFV_MCA_ indicated obvious cerebral hypoperfusion. Thus exposure to even mild +Gz hypergravity occasionally involves presyncope with cerebral hypoperfusion. In the other case (Fig. 3, *case 2*), both MCBFV_MCA_ and ET_CO2_ decreased remarkably during the last few minutes of +1.5 Gz centrifugation, suggesting that hyperventilation was induced. It was considered that the nausea and hyperventilation in this participant were induced by vestibular and/or other stimuli; however, we cannot clearly state that these decreases in MCBFV_MCA_ with hyperventilated hypocapnia have similar physiological meaning or significance to the obvious cerebral hypoperfusion with hypotension, such as presyncope. No simultaneous change in C-rSO_2_ was observed during this period in this participant; thus the relationship between C-rSO_2_ and CBF velocity under hyperventilated hypocapnia remains unclear.

In contrast to previous studies (15, 20, 24), we selected laymen who had no experience in a centrifuge as participants in the present study to detect physiological changes under mild hypergravity more clearly. As expected, many more data segments and indexes showed statistically significant differences in post hoc tests in the present study compared with previous studies. Although the standard deviation of MCBFV_MCA_ was relatively large, the individual trends of changes in MCBFV_MCA_ for each participant were similar (Fig. 1). The decreasing rates of MCBFV_MCA_ for the 0–5-min, 5–10-min, and 10–15-min data segments were smaller than those of MAP_MCA_. On the other hand, MAP_MCA_ tended to be restored in the latter half of +1.5 Gz centrifugation, but MCBFV_MCA_ was still decreasing, resulting in the greater decreasing rate of MCBFV_MCA_ (−7.4%) compared with MAP_MCA_ (−5.7%) for the 15–20-min data segment. This result suggested that cerebral autoregulation functioned during the early stages of mild +Gz hypergravity, but declined in the last stages of a 21-min centrifugation session in the laymen. However, it was thought that these small decreases in CBF did not lead to any symptoms, including cognitive deficits, in the present study. Moreover, in the present study, two of the participants could not accomplish the scheduled centrifugation protocol. Therefore, the physiological impacts of hypergravity for laymen seemed to be stronger than those for experienced participants, suggesting that the duration of centrifugation and more careful monitoring should be considered for individuals who do not have much experience with centrifuges, even if the magnitude of hypergravity is small.

There were some limitations in the present study. The possibility of changes in MCA diameter is a common limitation for studies using TCD. The changes in CBF were estimated by the changes in CBF velocity in the MCA based on the assumption that the MCA diameter does not change (1, 29). Recent studies have shown both dilation and constriction of the MCA by high-resolution MRI during higher levels of hypercapnia and hypocapnia, respectively (11, 12, 36). Therefore, the possibility of the constriction of the MCA due to significant decreases in ET_CO2_ during centrifugation in the present study could not be ruled out. However, the constriction of the MCA would cause the recorded CBF velocity to underestimate the actual CBF decrease. Thus it was thought that the possibility of changes in MCA diameter would not affect the present results of significant decreases in CBF, at least from the viewpoint of arterial blood gas; however, whether hypergravity would affect the MCA diameter remains unclear. In fact, the possibility that the MCA diameter would dilate under mild +Gz hypergravity also cannot be ruled out. If this dilation occurred, it could lead to normal or even elevated volume flow despite decreases in CBF velocity. In addition, if an increase in local blood volume just under the NIRS electrode occurred, it could also lead to the present result that C-rSO_2_ did not change despite decreases in CBF velocity. Moreover, since the relationship between CBF velocity and C-rSO_2_ under hyperventilated hypocapnia remains unclear, further study to evaluate this relationship using controlled breathing would be necessary. Another study evaluating the sensibility of C-rSO_2_ against changes in true hemoglobin saturation using mild hypoxic gas inhalation would also be useful. Furthermore, the participants in the present study had no experience with centrifuges before the experiment, and were much younger (24 ± 1 yr) than the astronauts recently participating in long-duration spaceflight (48.6 ± 4.7 yr) (26). Therefore, the findings in the present study might not be applicable to recent astronauts and future space travelers.

In conclusion, to test our hypothesis that C-rSO_2_ would decrease in association with a reduction in CBF, we evaluated simultaneously C-rSO_2_ and MCBFV_MCA_ during +1.5 Gz centrifugation. Contrary to our hypothesis, C-rSO_2_ did not change throughout the centrifugation, whereas MCBFV_MCA_ decreased significantly. In addition, no significant correlation was found between MCBFV_MCA_ and C-rSO_2_. The present results suggest that the necessary assumptions to monitor cerebral ischemia by cerebral oxygenation may not always be applicable, and cerebral oxygenation as measured by NIRS may not reflect decreases in CBF precisely under mild +Gz hypergravity. Thus measuring changes in CBF by NIRS may not be appropriate for the research setting. On the other hand, if the extent of decreases in CBF preponderate the impact of the violated necessary assumptions for NIRS, cerebral oxygenation might be able to detect decreases in CBF before the development of presyncope under even mild +Gz hypergravity, and therefore, might be useful for monitoring decreased CBF in clinical practice, such as in the field of aerospace medicine.

## GRANTS

This study was supported by MEXT KAKENHI Grant Number JP15H05939, which is a part of “Living in Space [Grant-in-Aid for Scientific Research on Innovative Areas (2015–2019)]”.

## DISCLOSURES

No conflicts of interest, financial or otherwise, are declared by the authors.

## AUTHOR CONTRIBUTIONS

T. Konishi, Y.O., and K.I. conceived and designed research; T. Konishi, T. Kurazumi, T. Kato, C.T., Y.O., and K.I. performed experiments; T. Konishi and K.I. analyzed data; T. Konishi, T. Kurazumi, T. Kato, C.T., Y.O., and K.I. interpreted results of experiments; T. Konishi and K.I. prepared figures; T. Konishi and K.I. drafted manuscript; T. Konishi, T. Kurazumi, T. Kato, C.T., Y.O., and K.I. edited and revised manuscript; T. Konishi, T. Kurazumi, T. Kato, C.T., Y.O., and K.I. approved final version of manuscript.
